# ABCB1 and ABCC11 confer resistance to eribulin in breast cancer cell lines

**DOI:** 10.18632/oncotarget.11727

**Published:** 2016-08-31

**Authors:** Takaaki Oba, Hiroto Izumi, Ken-ichi Ito

**Affiliations:** ^1^ Division of Breast, Endocrine and Respiratory Surgery, Department of Surgery (II), Shinshu University School of Medicine, Matsumoto, Japan; ^2^ Department of Occupational Pneumology, Institute of Industrial Ecological Sciences, University of Occupational and Environmental Health, Kitakyushu, Japan

**Keywords:** eribulin, drug resistance, breast cancer, ABCB1, ABCC11

## Abstract

This study aimed to elucidate the mechanisms underlying the resistance of breast cancer to eribulin. Seven eribulin-resistant breast cancer cell lines (MCF7/E, BT474/E, ZR75-1/E, SKBR3/E, MDA-MB-231/E, Hs578T/E, and MDA-MB-157/E) were established. mRNA and protein expression of ATP-binding cassette subfamily B member 1 (ABCB1) and subfamily C member 11 (ABCC11) increased in all eribulin-resistant cell lines compared to the parental cell lines. When ABCB1 or ABCC11 expression was inhibited by small interfering RNA in MCF7/E, BT474/E, and MDA-MB-231/E cells, eribulin sensitivity was partially restored. Moreover, eribulin resistance was attenuated additively by inhibiting ABCB1 and ABCC11 in MCF7/E cells. Additionally, overexpression of exogenous ABCB1 or ABCC11 in HEK293T cells conferred resistance to eribulin. MCF7/E and MDA-MB-231/E cells showed cross-resistance to paclitaxel, doxorubicin, and fluorouracil. Inhibition of ABCB1 partially restored paclitaxel and doxorubicin sensitivity. Partial restoration of fluorouracil sensitivity was induced by inhibiting ABCC11 in MCF7/E and MDA-MB-231/E cells. Both ABCB1 and ABCC11 are involved in the development of eribulin resistance in breast cancer cells *in vitro* regardless of the breast cancer subtype. Thus, ABCB1 and ABCC11 expression may be used as a biomarker for predicting the response to eribulin in patients with breast cancer. Concomitant inhibition of ABCB1 and ABCC11 might help enhance the antitumor effects of eribulin.

## INTRODUCTION

Eribulin mesylate (eribulin) is a synthetic macrocyclic ketone analog of the marine sponge natural product halichondrin B and is an inhibitor of microtubule dynamics [1, 2]. Eribulin inhibits microtubule polymerization by a mechanism distinct from those of other antitubulin agents such as vinblastine or taxanes. When administered to patients with metastatic breast cancer who had previously received both an anthracyclin and a taxane in either the adjuvant or metastatic setting, eribulin significantly increased their overall survival by monotherapy; thus, eribulin is currently an approved treatment for patients with advanced breast cancer [3]. However, most patients fail to respond to eribulin within several months, a phenomenon that has also been observed for many other cytotoxic drugs that were previously administered for breast cancer, and the mechanisms underlying this resistance to eribulin have not been fully elucidated.

ATP-binding cassette (ABC) transporters are primary active transporters that confer drug resistance by effluxing anticancer agents [4]. ABC transporters consist of 48 members that are classified into seven subfamilies from ABCA to ABCG based on their sequence similarity [5]. Among them, ABC subfamily B member 1 (ABCB1)/P-glycoprotein (*MDR1*) has been implicated as the major efflux transporter responsible for the resistance of cancer cells to many anticancer agents such as cisplatin, docetaxel, anthracyclines (doxorubicin, epirubicin), etoposide, irinotecan, methotrexate, paclitaxel, and vincristine [5, 6]. Members of the ABC subfamily C/multidrug resistance protein (ABCC/MRP) superfamily consist of 13 subfamily members (ABCC1 to ABCC13). Among the ABCC subfamily members, ABCC1 (MRP1) and ABCC2 (MRP2) are known to have transport mechanisms for a wide variety of chemotherapeutic agents; they also confer resistance to various anticancer agents including anthracyclines, etoposide, methotrexate, mitoxantrone, and cisplatin [5, 7–13], whereas ABCC11 confers resistance to fluorouracil, methotrexate, and pemetrexed [14–21]. However, the involvement of ABC transporters in the resistance of cancer cells to eribulin has not yet been reported.

To elucidate the mechanisms underlying the development of eribulin resistance in breast cancer cells, we established several eribulin-resistant cell lines using different breast cancer cell subtypes. In the present study, we demonstrated for the first time that ABCB1 and ABCC11 confer eribulin resistance in breast cancer cells regardless of their subtype.

## RESULTS

### Establishment of eribulin-resistant breast cancer cell lines

**Figure 1:**
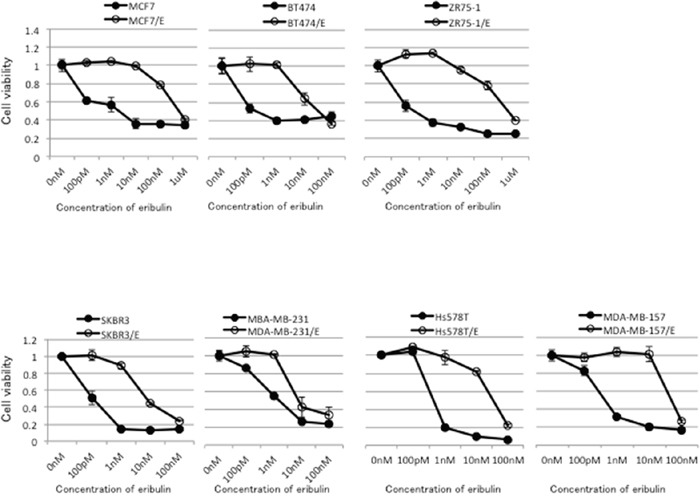
Sensitivity to eribulin in eribulin-resistant breast cancer cells and their parental cells Eribulin-resistant breast cancer cell lines were obtained by culturing MCF7, BT474, ZR75-1, SKBR3, MDA-MB-231, Hs578T, and MDA-MB-157 cells with stepwise-increasing doses of eribulin for more than 6 months. Sensitivity to eribulin was assayed by using the WST assay. Each cell line with “/E” indicates an established eribulin-resistant cell line. Closed circles (●) indicate parental cells, whereas open circles (○) indicate eribulin-resistant cells. The error bars represent the standard error of the value obtained in the experiments performed in triplicate.

Eribulin-resistant breast cancer cell lines were obtained by culturing MCF7, BT474, ZR75-1, SKBR3, MDA-MB-231, Hs578T, and MDA-MB-157 cells with stepwise increases in the eribulin concentration for more than 6 months. The eribulin-resistant cell lines were designated as MCF7/E, BT474/E, ZR75-1/E, SKBR3/E, MDA-MB-231/E, Hs578T/E, and MDA-MB-157/E, respectively. The relative eribulin resistance of each eribulin-resistant cell line compared to the corresponding parental cell line was determined using a WST assay (Figure 1). Although an eribulin-resistant cell line was established for each breast cancer cell line, the IC_50_ for the eribulin-resistant cell lines varied among the cell lines. The IC_50_ for the parental cell lines ranged from 0.1 to 1.3 nM, whereas the IC_50_ for the eribulin-resistant cell lines ranged from 4.7 to 556.7 nM. The eribulin resistance of the eribulin-resistant cell lines was 7.9-fold to 672.1-fold greater than that of the parental cell lines. The MCF7/E cell line showed the highest IC_50_ (556.7 ± 108.4 nM), whereas the SKBR3/E cell line showed the lowest IC_50_ (4.7 ± 0.4 nM).

The expression of estrogen receptor α (ERα) and human epidermal growth factor receptor-2 (HER2) in each parental cell line was evaluated by western blotting. Expression of ERα was detected in MCF7, BT474, and ZR75-1 cells. Expression of HER-2 was detected in BT474 and SKBR3 cells (Supplementary Figure 1). The characteristics of the parental cell lines and IC_50_ for eribulin in the parental and eribulin-resistant cell lines are summarized in Table 1.

**Table 1:** Characteristics and IC_50_ for eribulin in the parental and eribulin-resistant breast cancer cell lines

Cell line	MCF7		BT474		ZR75-1		SKBR3		MDA-MB-231		Hs578T		MDA-MB-157	
ERα	+		+		+		−		−		−		−	
HER2	−		+		−		+		−		−		−	
Parental or eribulin-resistant	P	E	P	E	P	E	P	E	P	E	P	E	P	E
IC_50_ (nM) (mean ± SD)	1.3 ± 0.5	556.7 ± 108.4	0.6 ± 0.1	20.8 ± 8.8	0.6 ± 0.1	403.3 ± 32.9	0.1 ± 0.1	4.7 ± 0.4	1.1 ± 0.1	8.7 ± 1.4	0.7 ± 0.0	26.2 ± 1.2	0.6 ± 0.0	56.7 ± 9.4
Relative resistance ratio^a^		428.2		34.6		672.1		47.0		7.9		37.7		94.5

P: parental cell, E: eribulin-resistant cell, IC_50_: half-maximal inhibitory concentration, ERα:estrogen receptor-α, HER2: human epidermal growth factor receptor type 2.

a Relative resistance ratio = IC_50_ of eribulin-resistant cells/IC_50_ of parental cells.

### Increased expression of ABCB1 and ABCC11 in eribulin-resistant breast cancer cell lines

**Figure 2:**
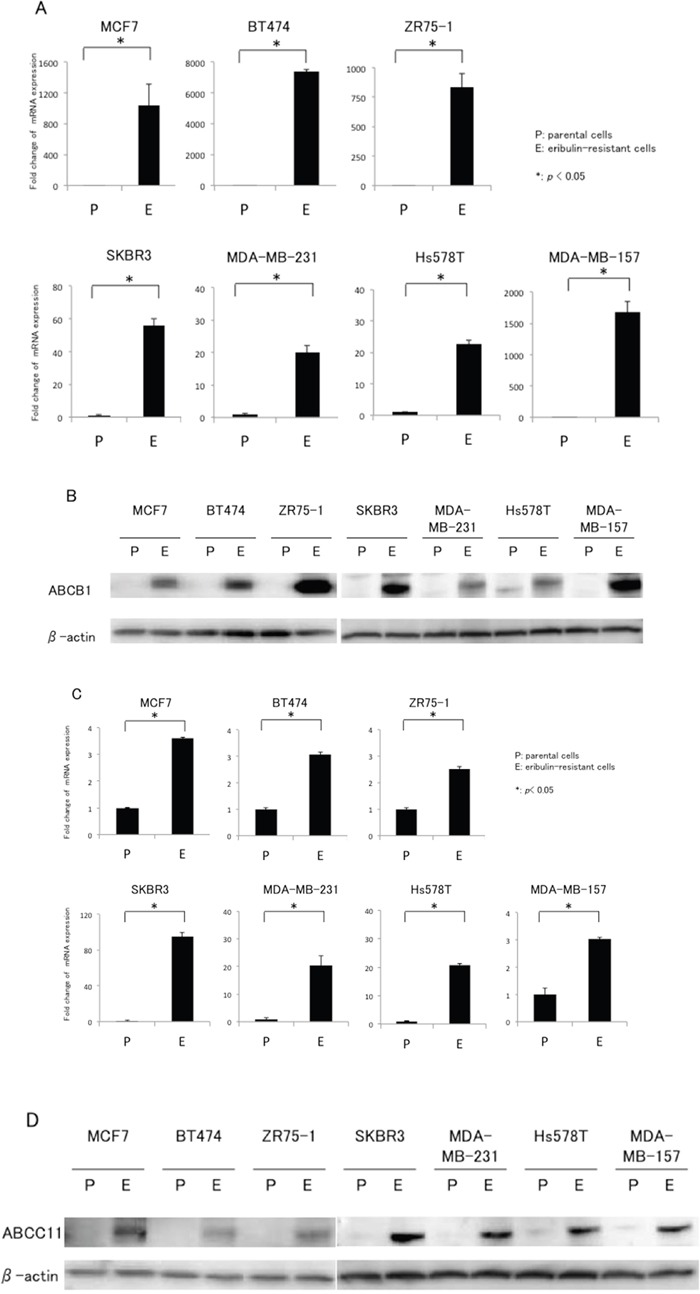
mRNA and protein expression of ABCB1 and ABCC11 in eribulin-resistant breast cancer cells and their parental cells **A.** ABCB1 mRNA expression quantitated by real-time RT-PCR. β-actin was used as an internal control. “P” indicates parental cell line and “E” indicates eribulin-resistant cell line. The error bars represent the standard error of the value obtained in the experiments performed in triplicate. **B.** ABCB1 protein expression analyzed with western blotting. β-actin was used as a loading control. **C.** ABCC11 mRNA expression quantitated by real-time RT-PCR. **D.** ABCC11 protein expression analyzed by western blotting. *P < 0.05 for parental cell line vs. eriburin-resistant cell line.

Prior to the present study, we compared the gene expression profile between MCF7 and MCF7/E cells by microarray analysis. The expression of ABCB1 and ABCC11 in MCF7/E cells increased 56.1-fold and 19.7-fold, respectively, compared to that in the parental MCF7 cells (data not shown). Based on this result, we examined the expression of ABCB1 and ABCC11 in seven breast cancer cell lines and their eribulin-resistant cells by real-time RT-PCR and western blotting. Figure 2A and 2B show the ABCB1 mRNA expression levels quantitated by real-time RT-PCR and the representative western blots of ABCB1, respectively, for the parental and eribulin-resistant cell lines. Although the expression levels varied from cell line to cell line, ABCB1 mRNA expression was significantly increased in all eribulin-resistant cell lines compared to the expression in the corresponding parental cell lines. In BT474/E, ZR75-1/E, and MDMB-157/E cells, a significant increase in ABCB1 expression was observed in both the real-time RT-PCR and western blot analyses, whereas the increased ABCB1 expression that was detected by real-time RT-PCR did not correspond to the results of the western blot analyses in the MCF7/E and SKBR3/E cells.

Figure 2C and 2D show the ABCC11 mRNA expression levels quantitated by real-time RT-PCR and representative western blots of ABCC11, respectively, for the parental and eribulin-resistant cell lines. Real-time RT-PCR revealed that expression of ABCC11 was significantly increased in all eribulin-resistant cell lines compared to the expression in the corresponding parental cell lines; moreover, the increases in ABCC11 expression that were detected by western blot analyses were similar to the expression increases observed in the real-time RT-PCR analyses. Hence, upregulation of both ABCB1 and ABCC11 in breast cancer cells was induced by continuous treatment regardless of the subtype of the cells.

### Restoration of eribulin sensitivity by ABCB1 or ABCC11 knockdown in eribulin-resistant breast cancer cells

**Figure 3:**
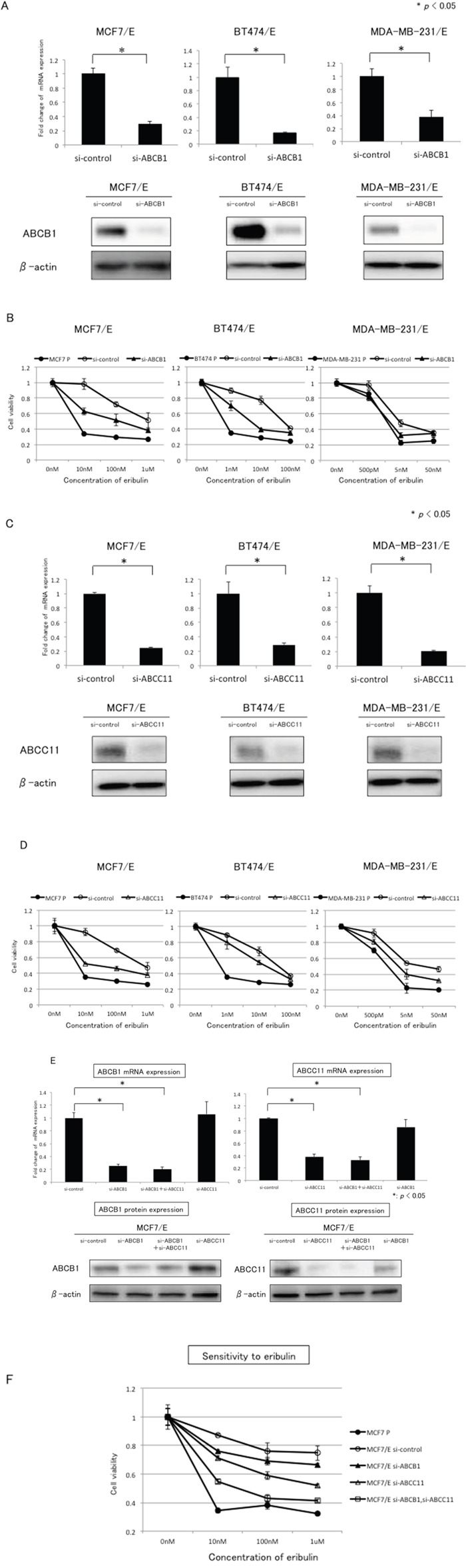
Effects of ABCB1 or ABCC11 knockdown in eribulin-resistant breast cancer cells The expression of ABCB1 and ABCC11 in MCF7/E, BT474/E, and MDA-MB231/E cells was inhibited by siRNA, and the sensitivity to eribulin was tested using WST assays. **A.** ABCB1 mRNA expression quantitated by real-time RT-PCR (upper panel) and representative results of western blot analyses (lower panel) in MCF7/E, BT474/E, and MDA-MB-231/E cells transfected with siRNA targeting ABCB1 (si-ABCB1) or control siRNA (si-control). *P < 0.05 for si-control vs. si-ABCB1. β-actin was used as a loading control. The error bars represent the standard error of the value obtained in the experiments performed in triplicate. **B.** Sensitivity to eribulin was measured in eribulin-resistant cells transfected with siRNA (si-control or si-ABCB1) and the parental cells. Closed circles (●) indicate parental cells, whereas open circles (○) indicate eribulin-resistant cells transfected with si-control; closed triangles (▲) indicate eribulin-resistant cells transfected with si-ABCB1, respectively. **C.** ABCC11 mRNA expression quantitated by real-time RT-PCR (upper panel) and representative results of the western blot analyses (lower panel) in MCF7/E, BT474/E, and MDA-MB-231/E cells transfected with siRNA targeting ABCC11 (si-ABCC11) or control siRNA (si-control). *P < 0.05 for si-control vs. si-ABCC11. β-actin was used as a loading control. **D.** Sensitivity to eribulin was measured in eribulin-resistant cells transfected with siRNA (si-control or si-ABCC11) and the parental cells. Closed circles (●) indicate parental cells, open circles (○) indicate eribulin-resistant cells transfected with si-control, and open triangles (D) indicate eribulin-resistant cells transfected with si-ABCC11. **E.** ABCB1 and ABCC11 mRNA expression quantitated by real-time RT-PCR (upper panel) and representative results of the western blot analyses (lower panel) in MCF7/E cells transfected with siRNA targeting ABCB1 (si-ABCB1), ABCC11 (si-ABCC11), both ABCB1 and ABCC11 (si-ABCB1 + si-ABCC11), or control siRNA (si-control). *P< 0.05. **F.** Sensitivity to eribulin was measured in the parental MCF7 cells and eribulin-resistant MCF7/E cells transfected with each siRNA. Closed circles (●) indicate parental MCF7 cells, open circles (○) indicate MCF7/E cells transfected with si-control, closed triangles (∆) indicate MCF7/E cells transfected with si-ABCB1, open triangles (∆) indicate MCF7/E cells transfected with si-ABCC11, and open squares (□) indicate MCF7/E cells transfected with both si-ABCB1 and si-ABCC11. The error bars represent the standard error of the value obtained in the experiments performed in triplicate.

To further examine the involvement of ABCB1 and ABCC11 in the development of eribulin resistance in breast cancer cells, we tested whether knockdown of ABCB1 or ABCC11 would restore eribulin sensitivity in eribulin-resistant breast cancer cells. We chose three eribulin-resistant cell lines (MCF7/E, BT474/E, and MDA-MB-231/E) for the experiment. Inhibition of ABCB1 expression by small interfering RNA (siRNA) was confirmed at both the mRNA and protein levels for the three cell lines (Figure 3A). Eribulin sensitivity was partially restored in MCF7/E and BT474/E cells, whereas siRNA targeting of ABCB1 resensitized the MDA-MB-231/E cells to eribulin to the same IC_50_ level as the parental MDA-MB-231 cells (Figure 3B).

Inhibition of ABCC11 expression by siRNA was confirmed at both the mRNA and protein levels in the three cell lines (Figure 3C). Eribulin sensitivity was partially restored in the three selected eribulin-resistant cell lines. The restoration effect was more potent in MCF7/E cells than in the other cells (Figure 3D). The growth inhibitory effects induced by eribulin in the eribulin-resistant cell lines in these experiments were of lower magnitude than the effects demonstrated in Figure 1, and this discrepancies was considered to result from the shorter incubation times in the siRNA experiments compared with the experiments used to determine the IC_50_ for each cell line.

Next, to evaluate whether the sensitivity to eribulin was restored additively by inhibiting expression of both ABCB1 and ABCC11 in the eribulin-resistant MCF7/E cells, knockdown of both ABCB1 and ABCC11 was performed in the MCF7/E cells. When the MCF7/E cells were transfected with siRNA that targeted ABCB1 and siRNA that targeted ABCC11, the expression of both ABCB1 and ABCC11 was inhibited to the same extent as when the cells were transfected with either siRNA alone. Expression of ABCB1 was not inhibited by transfection with the ABCC11-targeting siRNA, and expression of ABCC11 was not inhibited by transfection with the ABCB1-targeting siRNA (Figure 3E). Inhibition of both ABCB1 and ABCC11 expression in the MCF7/E cells restored eribulin sensitivity to approximately the level observed for the parental MCF7 cells (Figure 3F). These results demonstrate that inhibiting the expression of either ABCB1 or ABCC11 restored the sensitivity to eribulin, although the degree of restoration varied depending on the cell line, indicating that ABCB1 and ABCC11 are involved in the development of eribulin resistance in these breast cancer cells.

### Overexpression of either ABCB1 or ABCC11 confers resistance to eribulin in SV40-transformed human embryonic kidney (HEK293T) cells

**Figure 4:**
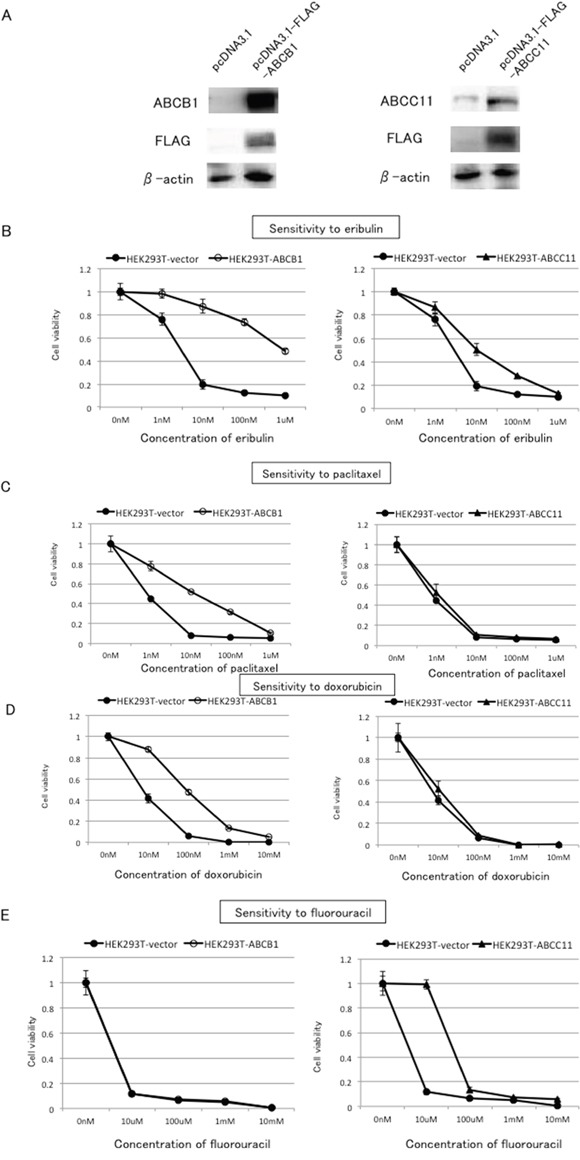
Effects of ABCB1 or ABCC11 overexpression on the sensitivity of HEK293T cells to eribulin, paclitaxel, doxorubicin, and fluorouracil A plasmid expressing Flag-ABCB1 (pcDNA3.1-FLAG-ABCB1) or Flag-ABCC11 (pcDNA3.1-FLAG-ABCC11) or an empty vector (pcDNA3.1) was transfected into HEK293T cells, and we tested whether overexpression of ABCB1 or ABCC11 altered the sensitivity to eribulin, paclitaxel, doxorubicin, and fluorouracil in the transiently transfected HEK293T cells. **A.** ABCB1 and ABCC11 protein expression in HEK-293T cells transfected with the plasmid DNA 24 h after transfection. Exogenously expressed ABCB1 and ABCC11 were detected by the specific antibodies for ABCB1 and ABCC11, and also the anti-FLAG antibody. The sensitivity to eribulin **B.** paclitaxel **C.** doxorubicin **D.** and fluorouracil **E.** in HEK293T cells transfected with the empty vector (HEK293T-vector) or the expression vectors for ABCB1 (HEK293T-ABCB1) or ABCC11 (HEK293T-ABCC11) was determined 72 h after transfection. Closed circles (●) indicate HEK293T-vector, open circles (○) indicate HEK293T-ABCB1, and closed triangles (▲) indicate HEK293T-ABCC11. The error bars represent the standard error of the value obtained in the experiments performed in triplicate.

To confirm that both ABCB1 and ABCC11 were able to confer resistance to eribulin in breast cancer cells, we tested whether transient overexpression of ABCB1 or ABCC11 would alter the sensitivity to eribulin in HEK293T cells. A plasmid containing Flag-ABCB1 or Flag-ABCC11 was introduced into HEK293T cells. HEK293T cells transfected with an empty vector (pcDNA3.1) were used as a control. In the HEK293T cells transfected with the empty vector, no ABCB1 expression was detected in western blots, whereas weak expression of ABCC11 was detected. Thus, neither ABCB1 nor ABCC11 was expressed at a high level in HEK293T cells. In contrast, high expression of ABCB1 and ABCC11 was detected in HEK293T cells that were transfected with the vectors expressing ABCB1 or ABCC11. The production of ABCB1 and ABCC11 by exogenously introduced genes was confirmed by immunoblots with an anti-FLAG antibody (Figure 4A).

In the HEK293T cells that were transfected with an empty vector, the IC_50_ for eribulin was 2.8 nM, whereas in the HEK293T cells that were transfected with ABCB1, the IC_50_ for eribulin was 833.3 nM, indicated a 297.6-fold in resistance compared to the cells transfected with the empty vector. In the cells that were transfected with ABCC11, the IC_50_ for eribulin was 9.1 nM, which indicated a 3.3-fold increase in resistance compared to the cells transfected with the empty vector. Thus, exogenous overexpression of both ABCB1 and ABCC11 was able to confer resistance to eribulin in HEK293T cells, although the degree of resistance induced by ABCB1 was more remarkable than that induced by ABCC11 (Figure 4B).

Furthermore, HEK293T cells that were transfected with ABCB1 became resistant to paclitaxel and doxorubicin, whereas HEK293T cells that were transfected with ABCC11 became resistant to fluorouracil (Figure 4C–4E). However, transfection of HEK293T cells with ABCC11 did not confer resistance to either paclitaxel or doxorubicin, whereas transfection of HEK293T cells with ABCB1 did not alter their sensitivity to fluorouracil (Figure 4C–4E).

### Cross-resistance to paclitaxel, doxorubicin, and fluorouracil in eribulin-resistant breast cancer cells

**Figure 5:**
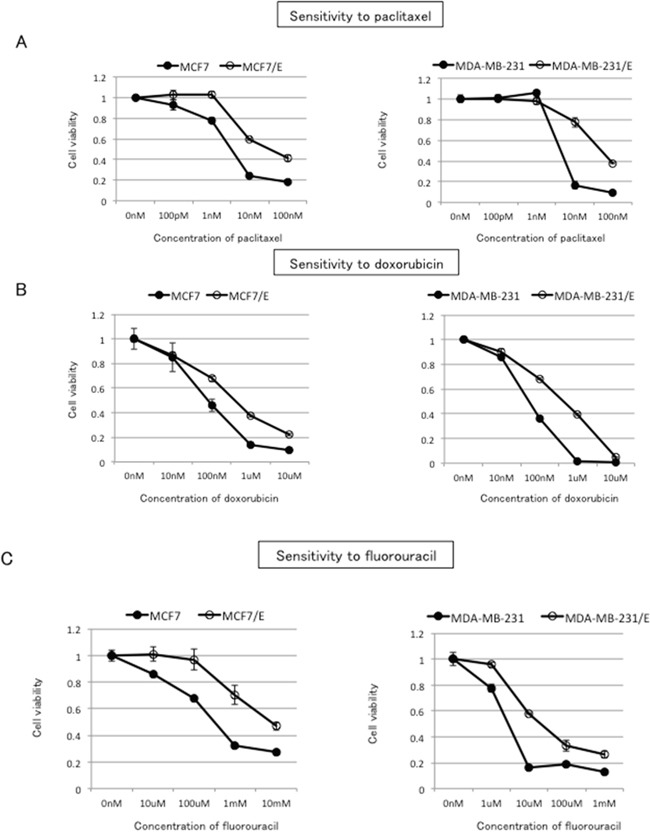
Cross-resistance to paclitaxel, doxorubicin, and fluorouracil in eribulin-resistant breast cancer cells Sensitivity to paclitaxel **A.** doxorubicin **B.** and fluorouracil **C.** in eribulin-resistant and parental MCF7 and MDA-MB231 cells was determined by WST assay. Closed circles (●) indicate parental cells, whereas open circles (○) indicate eribulin-resistant cells. The error bars represent the standard error of the value obtained in experiments performed in triplicate.

ABCB1 is known to confer resistance to a variety of anticancer agents including paclitaxel and doxorubicin [5]. ABCC11 is associated with resistance to fluorouracil [18]. In our experiments, overexpression of ABCB1 conferred resistance to doxorubicin, paclitaxel, and eribulin in HEK293T cells, whereas overexpression of ABCC11 conferred resistance to both fluorouracil and eribulin. Given these findings, we examined whether MCF7/E and MDA-MB231/E breast cancer cells would demonstrate cross-resistance to paclitaxel, doxorubicin, and fluorouracil. As demonstrated in Figure 5A, both MCF7/E and MDA-MB-231/E cells showed higher IC_50_ for paclitaxel, doxorubicin, and fluorouracil compared to their parental cell lines, indicating that both cell lines acquired cross-resistance to these three drugs in the course of developing resistance to eribulin (Figure 5 and Table 2).

**Table 2:** IC_50_ for paclitaxel, doxorubicin, and fluorouracil in parental/eribulin-resistant MCF7 and MDA-MB-231 cells

	MCF7	MCF7/E	RR	MDA-MB231	MDA-MB231/E	RR
Paclitaxel	2.1 ± 0.3 nM	29.3 ± 8.7 nM	13.9	4.1 ± 0.3 nM	50.3 ± 2.9 nM	12.3
Doxorubicin	79.3 ± 7.4 nM	376.7 ± 30.9 nM	4.8	50.3 ± 1.7 nM	453.3 ± 32.9 nM	9.0
Fluorouracil	306.7 ± 40.1 μM	4833.3 ± 154.5 μM	15.7	2.9 ± 0.3 μM	18.7 ± 0.5 μM	6.4

RR: relative resistance ratio = IC_50_ of eribulin-resistant cells/IC_50_ of parental cells.

### Inhibition of ABCB1 or ABCC11 alters the cross-resistance to paclitaxel, doxorubicin, and fluorouracil in eribulin-resistant breast cancer cells

**Figure 6:**
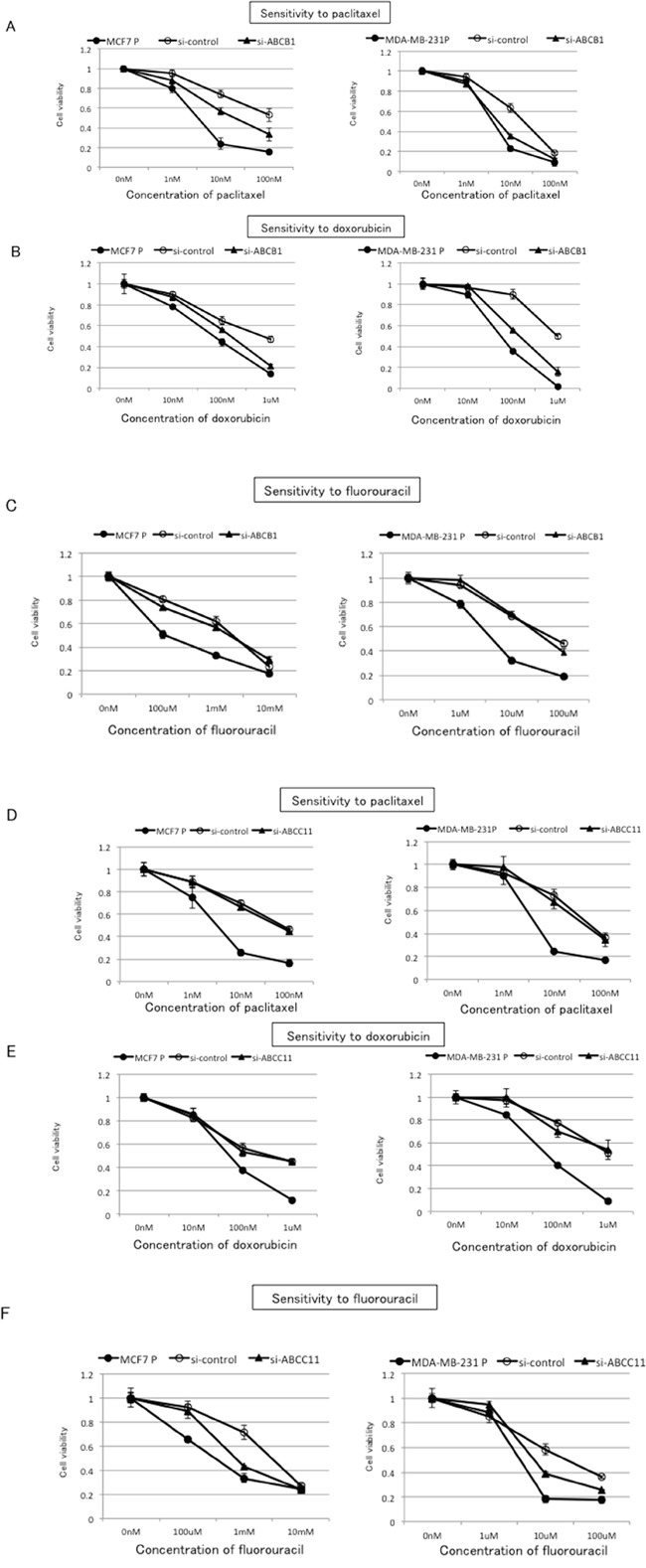
Effects of ABCB1 and ABCC11 knockdown on cross-resistance to paclitaxel, doxorubicin, and fluorouracil in eribulin-resistant breast cancer cells The expression of ABCB1 and ABCC11 in MCF7/E and MDA-MB231/E cells was inhibited by siRNA, and the sensitivity to paclitaxel, doxorubicin, and fluorouracil was tested by WST assay. Sensitivity to paclitaxel **A.** doxorubicin **B.** and fluorouracil **C.** in eribulin-resistant cells transfected with si-ABCB1 or si-control and the parental cells. Sensitivity to paclitaxel. **D.** doxorubicin **E.** and fluorouracil **F.** in eribulin-resistant cells transfected with si-ABCC11 or si-control and the parental cells. Closed circles (●) indicate parental cells, open circles (○) indicate eribulin-resistant cells transfected with si-control, and closed triangles (▲) indicate eribulin-resistant cells transfected with si-ABCB1 (A, B, and C) or with si-ABCC11 (D, E, and F), respectively.

To further investigate the involvement of ABCB1 and ABCC11 in the observed cross-resistance to paclitaxel, doxorubicin, and fluorouracil in MCF7/E and MDA-MB-231/E cells, we inhibited ABCB1 or ABCC11 expression with siRNA in these cells and evaluated the sensitivity to paclitaxel, doxorubicin, and fluorouracil (Figure 6). Both the mRNA and protein expression of ABCB1 and ABCC11 was decreased at 24 h after transfection with siRNA that targeted ABCB1 or ABCC11, respectively (Supplementary Figure 2). Inhibition of ABCB1 expression partially restored the sensitivity to paclitaxel and doxorubicin in both MCF7/E and MDA-MB-231/E cells (Figure 6A and 6B). Inhibition of ABCB1 expression did not alter the sensitivity to fluorouracil in either cell type (Figure 6C). In contrast, partial restoration of the sensitivity to fluorouracil was on inhibition of ABCC11 expression in MCF7/E and MDA-MB231/E cells, whereas inhibition of ABCC11 expression did not alter the sensitivity of either cell type to paclitaxel and doxorubicin (Figure 6D–6F). These results indicate that the cross-resistance to paclitaxel and doxorubicin that was observed in the MCF7/E and MDA-MB-231/E cells was mainly derived from ABCB1, whereas the cross-resistance to fluorouracil was mainly derived from ABCC11.

## DISCUSSION

Thus far, the mechanisms underlying resistance to eribulin have remained unknown. In the present study, we demonstrated that the expression of two ABC transporters, ABCB1 and ABCC11, which belong to different ABC transporter subfamilies, was increased in eribulin-resistant breast cancer cell lines. Furthermore, overexpression of ABCB1 and ABCC11 conferred resistance to eribulin independently in HEK293T cells. To the best of our knowledge, this is the first report to demonstrate the involvement of ABCC11 in the development of eribulin resistance in cancer cells. Although Laughney et al. [22] also reported an association between ABCB1 and eribulin resistance, our study is the first to show that resistance to eribulin can be directly induced by transfecting cells with the *ABCB1* gene *in vitro*. Our data suggest that both ABCB1 and ABCC11 are involved in the development of eribulin resistance in breast cancer cells and that eribulin or its metabolites might be substrates of ABCB1 and ABCC11.

ABC transporters play biologically important roles as membrane transporters [4]. In addition to their many physiological functions in the normal cells of various organs, some transporters have been known to induce resistance to anticancer agents by effluxing the agents themselves or their metabolites from cancer cells [23]. ABCB1 was identified in 1976 by Juliano and Ling [24] as a membrane glycoprotein that is overexpressed in colchicine-resistant cell lines and reduces drug permeability. ABCB1 has a wide substrate spectrum and can therefore mediate the export of a variety of drugs from different drug classes. These substrates include chemotherapeutic drugs such as anthracyclines, taxanes, and vinca alkaloids [6, 25–30].

In contrast, the classification of the ABCC subfamily originated from the finding of multidrug resistance protein-1 (MRP1) as the second major ABC drug transporter by Cole et al. in 1992 [13]. Three research groups cloned ABCC11, a member of the ABCC subfamily, independently in 2001 [15, 16, 20]. Subsequent studies demonstrated that a nonsynonymous single nucleotide polymorphism (538G>A) in the *ABCC11* gene determines the type of human earwax and axillary osmidrosis [31–33]. Additionally, it has been reported that human ABCC11 functions as an ATP-dependent efflux pump for amphipathic anions, including cyclic nucleotides, leukotriene C4, estrone 3-sulfate, estradiol 17-beta-D-glucuronide, and anti-viral agents [14, 19, 21, 34, 35]. Guo et al. [14] demonstrated that pig kidney epithelial cells transfected with wild-type ABCC11 exhibited increased resistance to fluorouracil, whereas increased resistance was not detected for vincristine, paclitaxel, doxorubicin, or etoposide. In lung cancer cell lines, ABCC11 has been reported to confer resistance to fluorouracil, methotrexate, and pemetrexed [14–21].

In our study, resistance to fluorouracil was induced by overexpression ABCC11 in HEK293T cells, whereas the sensitivity to paclitaxel or doxorubicin was not altered. Moreover, the sensitivity to fluorouracil was enhanced by inhibition of ABCC11 expression with siRNA in MCF7/E cells that demonstrated increased ABCC11 expression and cross-resistance to fluorouracil. Thus, our findings, together with the results of previous studies, demonstrate that ABCC11 is involved in the alteration of fluorouracil sensitivity in cancer cells. However, no previous studies reported an association between ABCC11 and resistance to eribulin. In the present study, increased ABCC11 expression was detected in all of the eribulin-resistant clones established by exposing seven different breast cancer cell lines to eribulin. In addition, exogenous expression of ABCC11 decreased the sensitivity to eribulin in HEK293T cells. Thus, it is clear that ABCC11 is involved in altering eribulin sensitivity in cancer cells. Although many molecules are likely involved in the development of eribulin resistance in clinical breast cancers, our report is the first to concretely reveal some of the mechanisms underlying eribulin resistance.

A meta-analysis of 31 studies previously demonstrated that ABCB1 (*MDR1*/P-glycoprotein) expression in tumors is associated with a poor response to chemotherapy [9–36]. Although the strategy to interfere with the transport activity of ABCB1 (*MDR1*/P-glycoprotein) has not been applied successfully in a clinical setting, accumulated data obtained from both *in vitro* and clinical samples indicate that ABCB1 (*MDR1*/P-glycoprotein) serves an important role in determining phenotype, including the susceptibility of breast cancer cells to anticancer agents [5, 6, 29, 36–38]. Ota et al. [39] reported an association between a single nucleotide polymorphism (538G>A) in ABCC11 and the risk of breast cancer in Japanese women. In addition, Yamada et al. [40] recently reported that ABCC11 was expressed significantly more frequently in aggressive subtypes and was associated with a poor prognosis in patients with breast cancer. These findings indicate a certain role of ABCC11 in the development and progression of breast cancer. It is intriguing that the expression of two ABC transporters with different substrate specificity was increased simultaneously by long-term exposure to eribulin in seven breast cancer cell lines, regardless of the receptor status of the cell lines. Eribulin binds to the vinca domain of tubulin; however, its antimitotic mechanism is distinct from that of other microtubule inhibitors such as vinca alkaloids and taxanes. Although the mechanisms underlying the transcriptional regulation of *ABCC11* have not been elucidated, a common transcriptional regulatory mechanism might exist for up-regulation of the *ABCB1* and *ABCC11* genes induced by eribulin-mediated stress.

The biology of breast cancer is known to depend largely on its subtype, which is determined mainly by the receptor status, and it is globally accepted that the therapeutic strategy is based on the subtype [41]. In the present study, upregulation of both ABCB1 and ABCC11 was observed in all seven breast cancer cell lines, regardless of their receptor status. These findings highlight the universal involvement of these transporters in the development of eribulin resistance in breast cancer cells. However, the degree to which ABCC11 contributed to eribulin resistance was much higher in MCF7 cells, which are positive for ERα, than in the triple-negative breast cancer cell lines. Thus, the degree to which each transporter contributes to eribulin resistance may vary among breast cancer subtypes. Honorat et al. [42] reported a positive correlation between ABCC11 and ERα in breast cancer cell lines and clinical specimens, and indicated a possible contribution of ABCC11 to the sensitivity to chemotherapeutic agents including fluorouracil in ERα-positive breast cancer that is resistant to tamoxifen. In the present study, the increased ABCC11 expression induced by the long-term exposure to eribulin conferred resistance to fluorouracil in ER-positive MCF7 cells. Therefore, our data strongly support that ABCC11 is involved in the development of fluorouracil resistance in ER-positive breast cancer cells.

Recently, Shi et al. demonstrated that ERα activates *ABCB1* transcription and increases paclitaxel resistance in ERα-positive breast cancer cells [43]. Their report together with the study of Honorat et al. described above suggests that ERα is involved in the regulation of both ABCB1 and ABCC11 expression in ERα-positive breast cancer cells, although these transporters were upregulated in breast cancer cells regardless of the expression of ERα in the present study. Therefore, further studies are required to elucidate the transcriptional regulation of these transporters in both ERα-positive and ERα-negative breast cancer cells.

Many anticancer agents have been applied in the treatment of breast cancer, and the prognoses of patients with recurrent or metastatic breast cancer have improved [44]. However, multidrug resistance induced by previously administered anticancer agents is frequently encountered in clinical practice and often limits the efficacy of subsequent anticancer agents. In the present study, both MCF7/E and MDA-MB231/E cells showed cross-resistance to three key drugs that are used to treat breast cancer, namely doxorubin, paclitaxel, and fluorouracil. Furthermore, the involvement of ABCB1 and ABCC11 in the cross-resistance was confirmed by siRNA-mediated knockdown. Although further examination is necessary to determine whether or not eribulin increases ABCB1 and ABCC11 expression in clinically treated breast cancer, the induction of resistance to key breast cancer treatment drugs should be taken into consideration when designing a treatment strategy for patients with recurrent breast cancer.

In conclusion, our study demonstrated that both ABCB1 and ABCC11 were able to confer resistance to eribulin; we also showed that increased expression of both ABCB1 and ABCC11 may be involved in the development of eribulin resistance in breast cancer cells, regardless of the subtype. Our results suggest that ABCB1 and ABCC11 may be used as biomarkers for predicting the response to eribulin in patients with breast cancer. Moreover, a novel therapeutic strategy for enhancing or prolonging the therapeutic effects of eribulin might be established by revealing the precise mechanisms underlying the induction of these transporters or by designing a specific inhibitor for them.

## MATERIALS AND METHODS

### Cell culture and agents

Seven breast cancer cell lines (MCF7, BT474, ZR75-1, SKBR3, MDA-MB-231, Hs578T, and MDA-MB-157) were purchased from the American Type Cell Collection (Manassas, VA) at the beginning of the study. HEK293T cells were kindly donated by Dr. Takeshita Toshikazu at Shinshu University. All cell lines were cultured in RPMI with 10% FBS at 37.0°C under 5% CO_2_. Eribulin-resistant cell lines were established in our laboratory by continuous exposure to stepwise increases in the concentration of eribulin for more than 6 months, during which time the medium was replaced every 3 days and the cultured cells were subcultured by trypsinization when they reached 70% confluency. Through this process, we selected several eribulin-resistant clones for each breast cancer cell line, and then used one representative clone in the subsequent experiments. The established eribulin-resistant clones were maintained in the eribulin-containing media throughout the experiments.

Eribulin was purchased from Eisai Co., Ltd. (Tokyo, Japan). Paclitaxel, doxorubicin, and fluorouracil were purchased from Sigma-Aldrich (Saint Louis, MO).

### WST assay

The growth inhibitory effects of eribulin, paclitaxel, doxorubicin, and fluorouracil were measured using a tetrazolium salt-based proliferation assay (WST assay; Wako Chemicals, Osaka, Japan) according to the manufacturer's instructions. Briefly, 4 × 10^3^ cells were cultured in 96-well plates in 100 μL of growth medium and incubated for 24 h. Then, 100 μL of medium with a graded concentration of eribulin, paclitaxel, doxorubicin, or fluorouracil was added into each well and cultured for 96 h in the experiment determining the IC_50_ for the eribulin-resistant cells or 72 h in the experiments involving transfection with siRNA or plasmid DNA. Then, 10 μL of WST-8 solution was added to each well, and the plates were incubated at 37°C for another 3 h. The absorbance was measured at 450 and 640 nm using SoftMax Pro (Molecular Devices, Tokyo, Japan), and the cell viability was determined. Each experiment was independently performed and repeated at least three times.

### Total RNA extraction and quantitative real-time RT-PCR

Total RNA was extracted using an RNeasy Mini kit (Qiagen, Alameda, CA) according to the manufacturer's instructions. TaqMan Gene Expression Assays for *ABCB1* (#Hs00184500_m1), *ABCC11* (#Hs01090768_m1), and β-actin (#Hs99999903_m1) were purchased from Applied Biosystems (Carlsbad, CA) and mRNA levels were quantified in triplicate using Applied Biosystems 7300 Real-Time PCR system (Carlsbad, CA).

### Microarray analysis

Total RNA from parental MCF7 cells and MCF7/E cells was extracted using an RNeasy Mini kit (Qiagen, Alameda, CA) according to the manufacturer's instructions. Microarray analysis using an Agilent SurePrint G3 Human GE v2 8x60K Microarray (Design ID: 039494) was performed at DNA Chip Research Inc. (Tokyo, Japan).

### Western blotting

Proteins were isolated from cells as previously described and used in western blot analyses (10 μg/lane) [36–37]. The membrane was probed with the following antibodies: anti-ERα antibody (1:200; Santa Cruz Biotechnology, Heidelberg, CA), anti-HER2 antibody(1:1000; Cell Signaling Technology, Danvers, MA), anti-ABCB1 antibody (1:1000; Cell Signaling Technology, Danvers, MA), anti-ABCC11 antibody (1:200; Santa Cruz Biotechnology, Heidelberg, CA), and anti-FLAG antibody (1:500; Sigma, Saint Louis, MO). β-actin (1:5000; Sigma, Saint Louis, MO) was used as a loading control. Each experiment was repeated independently at least three times and one representative blot was chosen for the figures.

### Transfection of siRNA

ON-TARGET plus siRNA for ABCB1 (L-003868), ABCC11 (L-004344), and the negative control (D-001810) were purchased from GE Healthcare (Buckinghamshire, England).

Transfection of each siRNA (10 nM) was performed using Lipofectamine RNAi-MAX (Thermo Fisher Scientific, Waltham, MA) following the manufacturer's instructions. Twenty-four hours after transfection, the total RNA or protein was extracted and 5 × 10^3^ cells/well were cultured in 96-well tissue culture plates and incubated for 72 h after addition of stepwise dilutions of eribulin, paclitaxel, doxorubicin, or fluorouracil. Finally, the absorbance was measured by using the WST solution as described above.

### Plasmid construction and transient transfection of expression vectors

To establish cells expressing Flag-tagged ABCB1, ABCC11, pcDNA3.1-FLAG-ABCB1 and pcDNA3.1-FLAG-ABCC11 were purchased from Genscript (Pistataway, NJ). Then, pcDNA3.1-FLAG-ABCB1, pcDNA3.1-FLAG-ABCC11, or pcDNA3.1 was transfected into HEK293T cells using FuGENE HD Transfection Reagent (Roche Diagnostics, Basel, Switzerland) following the manufacturer's protocol. An empty vector was included as a control in all experiments. Twenty-four hours after transfection, proteins were extracted and 5 × 10^3^ cells/well were cultured in 96-well tissue culture plates and incubated for 72 h after addition of stepwise dilutions of eribulin, paclitaxel, doxorubicin, or fluorouracil. Finally, the absorbance was measured by using the WST solution as described above.

### Statistical analysis

The levels of mRNA expression obtained by real-time RT-PCR were examined by Student's *t*-tests; *P*<0.05 was considered statistically significant (StatFlex ver.6, Artech Co., Ltd., Osaka, Japan).

## SUPPLEMENTARY FIGURES


