# Supplementary material for: ABCB1 and ABCC11 confer resistance to eribulin in breast cancer cell lines
Source: Oncotarget. 2016 Aug 31;7(43):70011–27. doi: 10.18632/oncotarget.11727 (PMC5342531; doi:10.18632/oncotarget.11727)
Supplement: Supplementary file 1 [file oncotarget-07-70011-s001.pdf]

ABCB1 and ABCC11 confer resistance to eribulin in breast cancer cell lines

SUPPLEMENTARY FIGURES

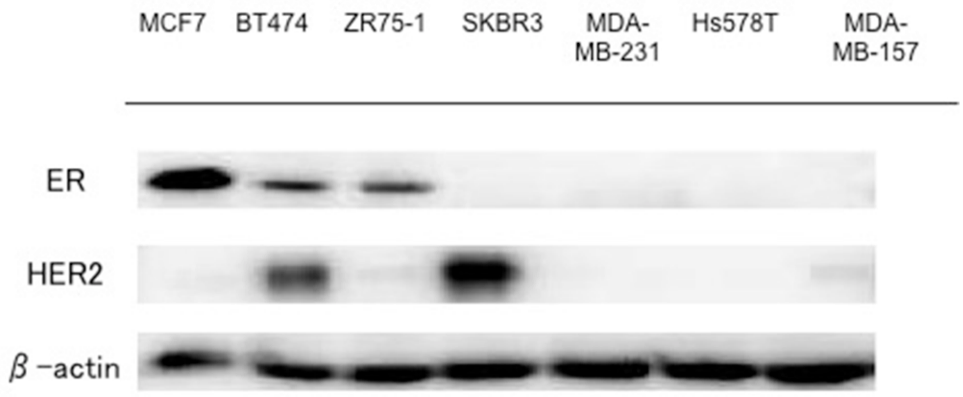

Supplementary Figure S1: The expression of estrogen receptor α (ERα) and human epidermal growth factor receptor-2 (HER2) in each parental cell line was evaluated by western blotting. β-actin was used as an internal control.

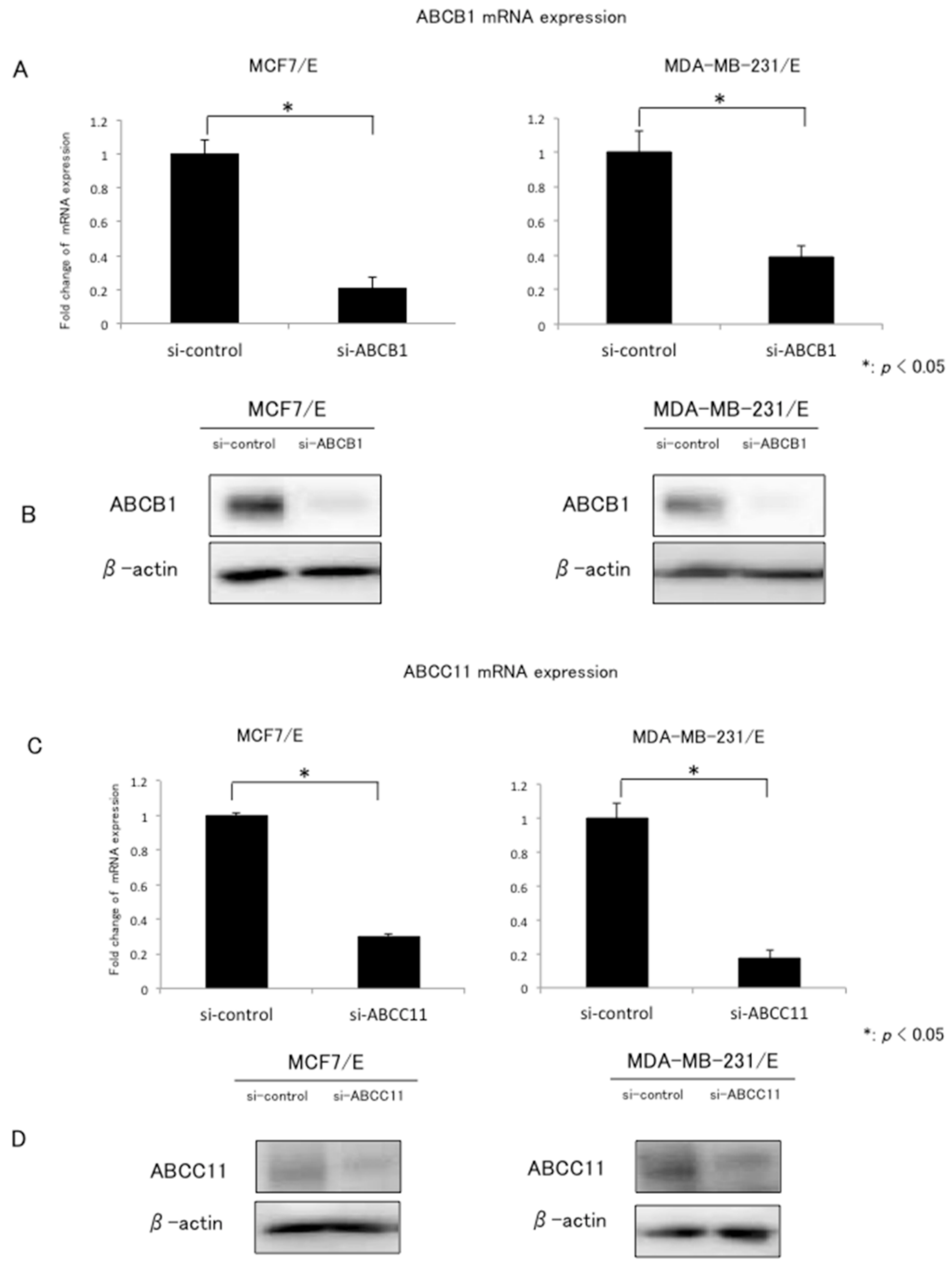

Supplementary Figure S2: Effects of ABCB1 or ABCC11 knockdown in eribulin-resistant breast cancer cells. The expression of ABCB1 or ABCC11 in MCF7/E and MDA-MB231/E cells was inhibited by siRNA. ABCB1 mRNA expression quantitated by real-time RT-PCR (A and C) and representative results of the western blot analysis (B and D) in MCF7/E and MDA-MB231/E cells transfected with siRNA targeting ABCB1 (si-ABCB1) or control siRNA (si-control). **P*<0.05 for si-control vs. si-ABCB1. β-actin was used as a loading control. The error bars represent the standard error of the value obtained in the experiments performed in triplicate
